# Former Primary Caregivers of Patients With Glioblastoma Multiforme Evaluate the PATH (Preparedness Assessment for the Transition Home) Instrument

**DOI:** 10.1111/jan.16420

**Published:** 2024-09-15

**Authors:** Elizabeth A. James, Marlee S. Wallace, Megan P. Chard, Michelle E. Camicia, Barbara J. Lutz, Laurie A. Minns

**Affiliations:** ^1^ Clinical Research, School of Nursing University of North Carolina Wilmington College of Health and Human Services Wilmington North Carolina USA; ^2^ Kaiser Foundation Rehabilitation Center, Kaiser Permanente Vallejo Medical Center Vallejo California USA

**Keywords:** brain, cancer, caregiver assessment, caregiver support, caregivers, case management, glioblastoma multiforme (GBM), newly diagnosed brain cancer patient, preparedness

## Abstract

**Aim:**

To evaluate whether Preparedness Assessment for the Transition Home (PATH), a validated instrument assessing gaps in caregiver commitment and capacity to care for a patient with a disabling condition, would be helpful to identify gaps in preparing primary caregivers of patients with glioblastoma multiforme (GBM).

**Design:**

A descriptive survey design with quantitative and qualitative data.

**Methods:**

Former primary caregivers of patients with GBM were invited to complete a 17‐question online survey during February and March 2023. Former caregivers, each having completed their caregiver journeys, are able to offer a unique perspective across the illness trajectory. Participants reviewed a copy of the PATH instrument and (a) responded to questions rating PATH helpfulness at each stage of the illness trajectory and (b) provided open‐ended feedback on the instrument.

**Results:**

One hundred seventeen of the 124 participants reported the PATH instrument would be helpful across all stages of the illness trajectory. While there were no statistically significant differences across the illness phases, response trends indicated using the PATH instrument earlier in the illness trajectory would have been more helpful to them as caregivers. Qualitative thematic analysis feedback indicated the most significant gap caregivers faced was education on the effects of the illness and treatment.

**Conclusion:**

It is vitally important to prepare and support caregivers. A validated instrument can identify unmet needs and inform care decisions.

**Implications for the Profession:**

Patient discharge plans should be guided by the needs and preferences of patients and caregivers. Identifying gaps in education and preparedness early in the illness trajectory may inform the care team of unmet needs, allowing them to tailor resources and support to improve outcomes for patients with GBM and their caregivers.

**Impact:**

Patient discharge plans should be guided by the needs and preferences of patients and caregivers. Identifying gaps in education and preparedness early in the illness trajectory may inform the care team of unmet needs, allowing them to tailor resources and support to improve outcomes for patients with GBM and their caregivers. PATH has the potential to inform healthcare professionals to develop customised care plans including education, resources and support for caregivers and patients with life‐threatening illness.

**Reporting Method:**

Study adheres to the STROBE reporting method.

**Patient or Public Contribution:**

Prior to deploying the survey to study participants, in addition to testing by study collaborators (authors), the survey was tested and feedback was received from graduate students and from administrators of the private Facebook group where the survey was promoted to study participants.

**Summary**

- Application of a validated caregiver assessment to identify and address gaps in understanding and preparation for caregivers of patients with glioblastoma multiforme and other debilitating illnesses.
- Highlight the limited participation of important healthcare domains such as mental health and palliative care in caregiving.

## 1 Introduction

Glioblastoma multiforme (GBM) is the most common type of primary malignant brain tumour with an incidence of 3.19 per 100,000 cases in the United States and a median survival of 15 months (Davis 2016; Thakkar et al. 2014). There is no known cure for GBM and it is nearly twice as common in men compared to women, with the median age of diagnosis at 64 years (Davis 2016). The current standard of care involves maximal surgical resection followed by concurrent temozolomide chemotherapy and radiation to slow illness progression and manage symptoms (Stupp et al. 2005). Due to the tumour location in the brain, patients' physical, mental and psychological functioning change significantly during the illness progression (Thakkar et al. 2014).

Neurological symptoms experienced by patients with GBM require constant care and support typically provided by an informal caregiver, such as a family member without nursing or medical training, who helps with activities of daily living, physical care and emotional support (Hickey 2019). Furthermore, the rapid and irreversible illness progression combined with cognitive decline in patients with GBM leaves caregivers with unique time‐sensitive challenges often inadequately addressed by the medical community (Boele et al. 2014; McConigley et al. 2010; Renovanz et al. 2017; Seekatz et al. 2017). Among the physical challenges of caregiving are helping patients with GBM navigate their symptoms; for example, 25% of patients experience seizures prior to diagnosis and more than 50% experience seizures in later phases of the illness (Davis 2016). Caregivers of patients with GBM expressed a need for better education from healthcare team members during each of the three stages of the illness progression: ‘the acute phase’ after initial diagnosis and standard of care measures; the ‘living with illness progression phase’ and the ‘end of life phase’ (Coman et al. 2024).

The illness trajectory of patients with GBM is largely defined by the medical care received in each phase because patient status and caregiver role vary greatly (Coman et al. 2024). For example, a sudden role shift from spouse/partner to caregiver can lead to significant burdens associated with caregiving (Coman et al. 2024). During the acute phase, medical visits are frequent, may last all day and may require multiple trips per week; this phase typically lasts 2–3 months. Caregivers, as the advocates for their loved one, reported high levels of stress during this intense phase. Self‐care is often deprioritised, and caregivers may experience adverse health events (Coman et al. 2024; Philip et al. 2015). Caregiver shock and inability to process the changes the patient is undergoing also occurs during this phase (Coman et al. 2024). Caregivers express the highest level of distress and the greatest need for educational support during the illness progression phase when medical visits decrease and the patient is typically on monthly maintenance temozolamide, where the patient receives temozolamide on days 1–5 during a 28‐day cycle that can last between 6 and 12 months (Huang, Yu, and Liang 2021). As the patient enters the end‐of‐life phase, functioning across all faculties declines rapidly. The primary caregiver at this stage has increased emotional needs preparing for the passing of their loved one, and the physical needs of the patient extend beyond what the primary caregiver and family can manage. Hospice or nursing care is often needed to help meet the patient's physical needs during this time (Choi et al. 2012; Piil et al. 2018). Two‐thirds of patients with GBM die at home (Coman et al. 2024; Heese et al. 2013).

Following the death of the patient, caregivers begin the bereavement phase. Processing their experience and reflecting on the life of their loved one is a key theme. Bereavement has no time limit and caregivers report life‐impacting effects including post‐traumatic stress disorder (Coman et al. 2024).

The sudden onset of GBM followed by the need for rapid medical interventions and decision‐making can propel someone into the role of caregiver without warning, often within days of diagnosis. Medical information and terminology are likely beyond caregivers' understanding (Sherwood et al. 2016). Comprehending the actual diagnosis and its impacts on the patient, caregiver and family are challenging and overwhelming. Patients are often sent home without adequately equipping the caregiver to handle care at home (Adelman et al. 2014). Caregivers are desperate for information and training and often turn to internet searches, where they might identify advocacy and support groups (Coman et al. 2024). Caregivers want to be included in care teams in which a family centred approach would provide better support for all concerned (Coman et al. 2024). Coping with the diagnosis, learning how to provide care and preparing for and navigating bereavement are significant gaps in preparing caregivers of patients with GBM.

Increasing readiness through assessment and individualised intervention has a direct effect on supporting caregivers so that they can provide better care for the person living with GBM (Roth, Preusser, and Weller 2016). Identifying and addressing these gaps may help fill unmet needs with the objective of increasing caregiver readiness (Camicia et al. 2022). The Preparedness Assessment for the Transition Home (PATH) instrument was initially validated for caregivers of stroke patients to assess their commitment, capacity and understanding of the long‐term implications of the illness. The PATH instrument is a 25‐question multiple choice instrument. Sample questions from the PATH instrument are provided (Table 1).

**TABLE 1:** PATH instrument example questions.

‘How much do you understand about the patient's expected recovery in the next 6 months?’ ‘How much do you understand about how the patient's injury/illness will affect your lives in the next 6 months?’ ‘How much do you understand about what you need to do to get things ready before the patient goes home?’

## 2 Background

The PATH instrument evaluates three areas that may be very helpful for caregivers of GBM patients including: (a) conducting a risk assessment of both patient and caregiver, (b) identifying gaps between the patient's needs and caregiver's capacity and commitment and (c) developing a customised plan for improving caregiver readiness based on results of the risk assessment (Camicia 2015; Camicia, Ann Laslo, and Lutz 2021). Following its use by caregivers of patients transitioning home after rehabilitation for stroke, the PATH instrument was updated to remove illness‐specific references, allowing for expanded use by caregivers of persons with a debilitating illness (Camicia, Lutz, Summers, et al. 2021; Camicia and Lutz 2016; Camicia et al. 2019, 2020; Camicia, Ann Laslo, and Lutz 2021). The purpose of this study was to invite former primary caregivers of patients with GBM evaluate the PATH instrument and provide feedback on whether it would have been helpful for them during the phases of GBM and to capture caregiver experiences. Former caregivers of patients with GBM were recruited to capture experience throughout the entire disease trajectory.

## 3 The Study

The overall aim of the study was to evaluate the PATH instrument for its helpfulness based on feedback from former primary caregivers of patients with GBM during the different phases of the illness trajectory and to provide feedback on the instrument via open‐ended questions.

Four research questions were addressed:

1. Will the PATH instrument be helpful to caregivers of patients with GBM?
2. At which stage(s) of the illness trajectory (acute, living with illness progression, end‐of‐life) will the PATH instrument be the most helpful to GBM caregivers?
3. Should the existing questions in the PATH instrument be revised to be more useful to GBM caregivers?
4. What new questions should be added to the PATH instrument to improve its helpfulness to GBM caregivers?

A secondary aim was to learn about the composition of the team involved in the overall care of patients with GBM and where patients received care. The intent of capturing this data was to gain knowledge of the unique experiences of GBM caregivers and to identify resource gaps.

## 4 Methodology

### 4.1 Design

A descriptive survey study design was employed with quantitative and qualitative data. Permission to use the PATH instrument for this study was received from the PATH authors. The survey captured demographic information and provided a copy of the PATH instrument for participants to review for rating the helpfulness of PATH using Likert‐type questions and open‐ended questions for qualitative reflexive thematic analysis. The study questionnaire and scale were developed as part of the study. The reference instrument PATH is an existing tool that has been validated for stroke patients. The primary purpose of the study was to determine whether the PATH instrument would be helpful to primary caregivers of patients with GBM. The quantitative part of the study consisted of questions rating helpfulness at each stage of the illness trajectory and overall helpfulness of the PATH using a Likert‐type scale. Open‐ended feedback was analysed using thematic qualitative analysis to inform future research into the impact of how tailored plans and/or interventions can improve caregiver readiness. The study was conducted with prior caregivers of patients with GBM in the United States.

### 4.2 Sample and Study Setting

Study participants included members of private Facebook group, ‘We are the Widows and Widowers of GBM’ (2.5K members, March 30, 2015; Coman et al. 2024). At the time of the study, the group had 2246 registered members and is a group for widows/widowers. This group includes legal spouses, long‐term partners and fiancé' relationships. We selected this group as a convenience sample based on members of the group being active in caregiving discussions and because the online group captures more potential participants than we could approach at individual medical centres based on GBM being a rare disease. L.A.M. is a former caregiver of a patient with GBM who posted the research study in the Facebook group and asked members to participate. For this study, participants reviewed the PATH instrument and provided feedback on whether they perceived that the instrument would have been helpful to them as a caregiver of a patient with GBM using a Likert scale. Individuals who agreed to complete the survey were also promoted to share open‐ended feedback on suggested improvements to the instrument for the GBM caregiver population. Prior to deploying the questionnaire, it was tested by study collaborators including undergraduate and graduate students and administrators of the Facebook group.

### 4.3 Inclusion/Exclusion Criteria

To be included in the study, participants must (1) be aged 18 or over; (2) have been a primary caregiver, spouse, partner or other life partner of a patient with GBM; (3) be a member of the Facebook group ‘We are the Widows and Widowers of GBM’ and (4) have provided informed consent by proceeding with the survey (Table 2). Exclusion criteria were (1) participants who did not complete informed consent, (2) participants residing outside of the United States and (3) any active caregiver of a patient with GBM. Both female and male caregivers were included if the following inclusion/exclusion were met. Refer to the study promotion text posted on the Facebook page (Table 3). Since GBM is a rare disease and patient experiences may vary widely based on the type of facility and resources available where care was received, we specifically recruited patients through the Facebook support group in an attempt to reflect the caregiver experience regardless of the type of medical facility and the resources offered.

**TABLE 2:** Study consent.

Your participation is voluntary. You may refuse to participate or may refuse to answer any question. You may stop at any time without penalty. Proceeding to complete the survey indicates consent to participate

**TABLE 3:** Study promotion.

Community Members, A graduate student interested in evaluating a tool to better equip GBM caregivers has invited our community to participate in an online survey. The tool has been used for stroke patients and for loved ones with other debilitating diseases and we hope it can make a meaningful difference for our community The goal of the survey is to evaluate if the Preparedness Assessment for the Transition Home (PATH) Instrument is appropriate to determine the readiness of primary caregivers of patients with GBM. The study will also explore at what time points within the GBM caregiver journey the tool could provide the greatest benefit to caregivers To learn more: <link to study participant letter> The survey will take less than 15 min to complete and was approved by the UNCW IRB #H22‐0103

### 4.4 Data Collection

Both quantitative and qualitative data were collected from the questionnaire. Quantitative demographics and helpfulness ratings using Likert‐style questions were captured. Qualitative data were captured by open‐ended questions asking respondents to provide feedback on the PATH and suggest changes to improve the instrument for use by GBM patients and caregivers. The study questionnaire (Data S1) was designed in collaboration with co‐authors, a prior caregiver (L.A.M.) and a co‐developer (B.J.L., a registered nurse) of the PATH instrument and administrators of the Facebook group. L.A.M. posted the survey three times. The questionnaire was developed in Qualtrics, a commercially available web‐based tool, and deployed between February 10, 2023 and March 1, 2023 following approval from the Facebook group administrators. All responses were anonymous and all data received were stored in a password‐protected computer system with access limited to co‐authors. Descriptive statistics were all categorical and aggregated for reporting using counts and percentages.

### 4.5 Qualitative Analysis: Reflexive Thematic Analysis

The qualitative component of the study assessed participant feedback on the PATH instrument with the following two questions: (1) do you have any suggestions to revise the existing questions in the PATH tool to be more helpful to GBM caregivers and (2) do you have any suggestions for new questions to add to the PATH tool to improve the helpfulness to GBM caregivers? Response options were (1) yes (if yes, please provide feedback) and (2) no. Responding to the qualitative questions was optional. Seventy‐five free text comments were captured recommending improvements to the PATH instrument for use in the GBM caregiver population. The comments varied in length from 4 words to 146 words. Comments were coded by research team members and were analysed using thematic qualitative analysis as described by Braun and Clarke (2019). The principal investigator (PI) in this study is a member of the Facebook group and has personal experience as a caregiver of a spouse with GBM; thus, the PI's active role in knowledge production is acknowledged and highlighted (Braun and Clarke 2019). Co‐investigators included students and an expert in qualitative and caregiving research. Responses were shared with the research group for review and familiarisation; the team then held three sessions to review, discuss and code the feedback. The workgroup adjudicated any areas in which members interpreted the feedback differently. After all codes were captured, they were reviewed for themes.

### 4.6 Ethical Considerations

To protect study participants, the study enrolled only former (not current) caregivers of patients with GBM. Both females and males were included if all inclusion/exclusion criteria were met. This study was reviewed by the UNCW IRB and was determined to be exempt according to the regulatory category 45 CRF46.104(d), category 02 (IRB#H23‐0583). Upon opening the survey, participants were asked to consent to the research.

## 5 Results

### 5.1 Demographic Characteristics of Participants

Of the 199 potential subjects who opened the recruitment post, eight did not consent to participate. Therefore, 191 participants consented and completed the demographic section. Of the 191 participants, 67 did not respond to the survey and the remaining 124 completed the survey questions in their entirety. Of the 124 complete quantitative responses, 42 participants responded to the optional feedback questions. Summary statistics for demographics included all who completed the demographic questions regardless of whether they fully completed the survey or not. The decision to include demographic data collected was based on a (1) high number of dropouts (67) and (2) research interest to better understand where treatment was received and care team members involved with each caregiver/patient dyad. The age range of study (caregiver) participants was 25–73 years (median: 54 years). The age range of GBM patients at the time of diagnosis was 27–77 years (median: 57 years). Study participants reported the length of their bereavement as ranging from 0.5 to 146.0 months (median duration: 25 months). In this study, 97.9% (*n* = 187) of study participants were female, 0.5% (*n* = 1) were male and 0.5% (*n* = 1) were other gender. Study participants reported that 99% (*n* = 189) of patients were male, and 97.4% were a spouse or partner of the patient (Table 4).

**TABLE 4:** Demographics.

Demographics	*n* = 191	%
Participant age at diagnosis		
<30 years	2	1.0
30–39 years	18	9.4
40–49 years	40	20.9
50–59 years	74	38.7
60–69 years	48	25.1
70–79 years	8	4.2
Missing	1	0.5
Patient age at diagnosis		
<29 years	1	0.5
30–39 years	11	5.8
40–49 years	32	16.8
50–59 years	69	36.1
60–69 years	59	30.9
70–79 years	19	9.9
Participant gender		
Female	187	97.9
Male	1	0.5
Other	1	0.5
Missing	2	1.0
Patient gender		
Female	0	0.0
Male	189	99.0
Missing	2	1.0
Relationship		
Married or partner	186	97.4
Divorced or separated	1	0.5
Parent	1	0.5
Missing	3	1.6
Bereavement months		
0.5–0.12 months	54	28.3
13–24 months	38	19.9
25–36 months	43	22.5
37–48 months	23	12.0
49–56 months	11	5.8
57–64 months	6	3.1
>65 months	13	6.8
Missing	3	1.6
Received rehab		
Yes	152	79.6
No	36	18.8
Missing	3	1.6
Rehab: Acute stage		
Yes	95	49.7
No	93	48.7
Missing	3	1.6
Rehab: Disease prog. stage		
Yes	94	49.2
No	94	49.2
Missing	3	1.6
Rehab: End‐of‐life stage		
Yes	42	22.0
No	146	76.4
Missing	3	1.6

**FIGURE 1:**
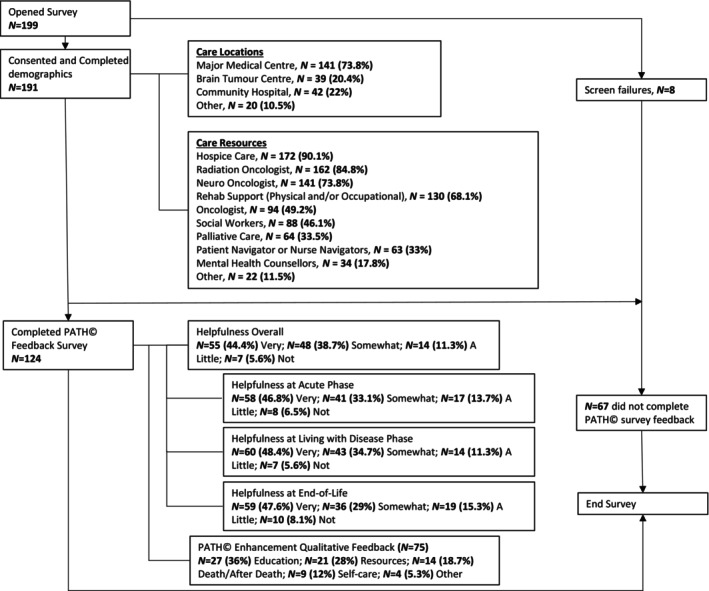
Study participant summary.

Sixty‐seven (35%) participants dropped out after completing demographics or reviewing the PATH instrument and did not return to complete the survey questions. A total of 124 participants provided feedback on the helpfulness of the PATH instrument (Figure 1) and this sample size was used for the main data analysis to assess the helpfulness of the PATH instrument. Those who responded to the survey questions reported slightly longer bereavement periods (26 months vs. 23.5 months); however, this difference was not statistically significant and no other major differences in demographic data were found between those who provided feedback about the PATH instrument and those who did not (Table 5).

**TABLE 5:** Demographic characteristics of PATH survey responders versus non‐responders.

	Responded to PATH survey (*n* = 124)	Did not respond to PATH survey (*n* = 67)
Participant age at Dx	Range = 24–73 years Median = 54 years	Range = 32–72 years Median = 54 years
Patient age at Dx	Range = 27–77 years Median = 57 years	Range = 38–74 years Median = 56.5 years
Bereavement months	Range = 0.5–146 months Median = 26 months	Range = 1–108 months Median = 23.5 months
Community medical centre	10.5% (*n* = 13 patients)	10.4% (*n* = 7 patients)
Major medical centre	89.5% (*n* = 111 patients)	89.6% (*n* = 60 patients)

### 5.2 Participant Evaluation of the PATH Instrument and Its Helpfulness During the Caregiver Journey

**FIGURE 2:**
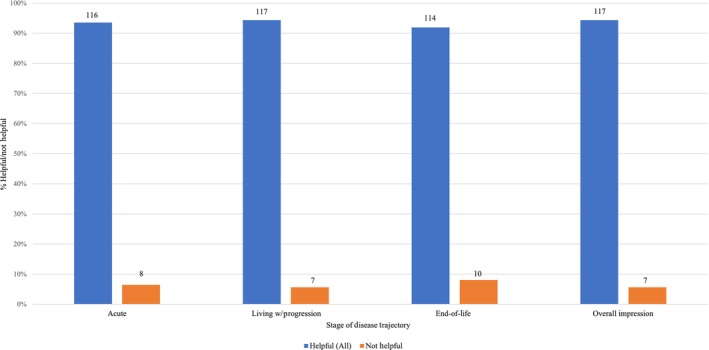
Participants report the PATH instrument would have been helpful overall.

**FIGURE 3:**
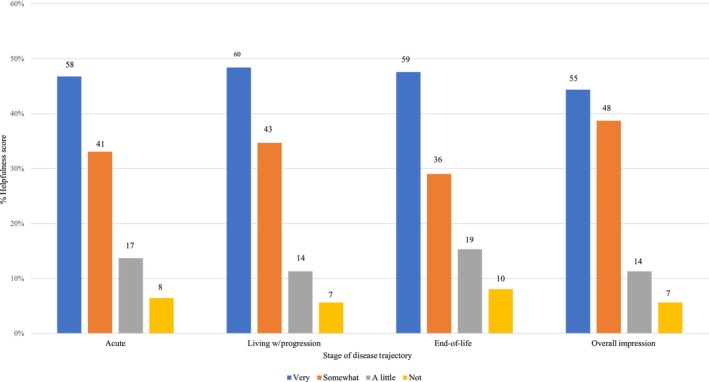
Participants report the PATH instrument would have been helpful throughout the disease trajectory.

Study participants overwhelmingly indicated that the PATH instrument would have been helpful to them across all phases of the illness trajectory (Figure 2), with most (83.1%; *n* = 103) responders selecting ‘very’ or ‘somewhat’ helpful overall: (1) 44.4% (*n* = 55) reported their overall impression for helpfulness as ‘very’ helpful; (2) 38.7% (*n* = 48) selected ‘somewhat’ helpful overall; (3) 11.3% (*n* = 14) selected ‘a little’ helpful overall and (4) 5.6% (*n* = 7) selected ‘not’ helpful overall. (Figure 3) Six per cent (*n* = 8) of participants did not think the instrument would be helpful at the ‘acute’ phase and 5.6% (*n* = 7) did not think the instrument would be helpful at the ‘living with illness progression’ phase. Approximately, 8% (*n* = 10) did not think the instrument would be helpful at the ‘end‐of‐life’ phase (Figure 2). Helpfulness data were collected overall and at each illness phase. Results are based on the 124 participants who completed the full survey.

### 5.3 Type of Medical Center Where Treatment Occurred

**FIGURE 4:**
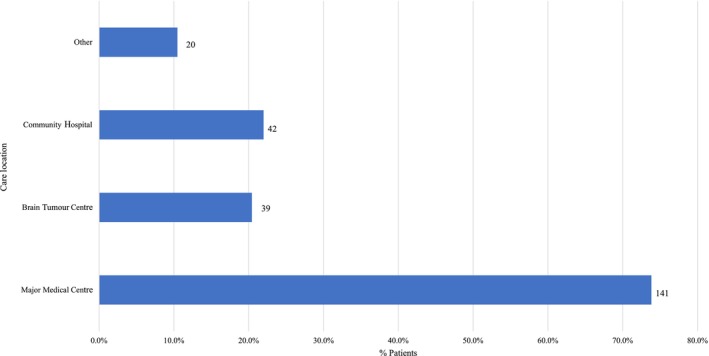
Most patients received care at major medical centres.

Since patient and caregiver resources may be different depending on the setting in which patients receive care, we asked participants to indicate where patients received treatment and illness management; respondents could select ‘all that apply’ from major medical centres, brain tumour centres, community hospitals and others. One hundred forty‐one (73.8%) participants indicated patients received care at a major medical centre, 39 (20.4%) at a brain tumour centre, and 42 (22.0%) at a community hospital; 20 (10.5%) participants reported receiving care an ‘other’ type of facility (Figure 4). Medical centre data are based on the 191 participants who completed demographics.

### 5.4 Care Team Members

**FIGURE 5:**
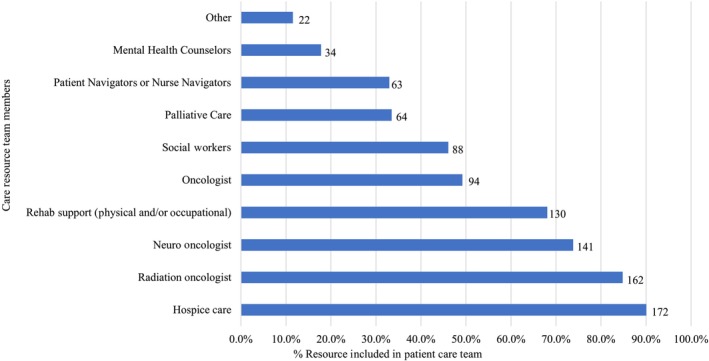
Patient care team.

Since cancer care requires interdisciplinary collaboration among healthcare team members, participants indicated who was part of the patient's care team; respondents selected all care resources used during the patient's illness and end‐of‐life including: mental health counsellors, patient navigators or nurse navigators, palliative care, social workers, oncologists, rehabilitation support (physical and/or occupational therapy), neurooncologists, radiation oncologists and hospice care. Caregivers reported the highest levels of engagement with hospice care (*n* = 172, 90.1%), radiation oncologists (*n* = 162, 84.8%), neurooncologists (*n* = 141, 73.8%) and physical and/or occupational rehabilitation support (*n* = 130, 68.1%). Less frequently 94 caregivers (49.2%) reported receiving care from an oncologist and 88 (46.1%) from a social worker. The least used and/or available care resources included: palliative care (*n* = 64, 33.5%), patient navigators and/or nurse navigators (*n* = 63, 33.0%) and mental health counsellors (*n* = 34, 17.8%) (Figure 5). Care team member data are based on the *n* = 191 who completed demographics.

### 5.5 Recommendations to Modify the PATH Instrument for Patients With GBM and Their Caregivers

**FIGURE 6:**
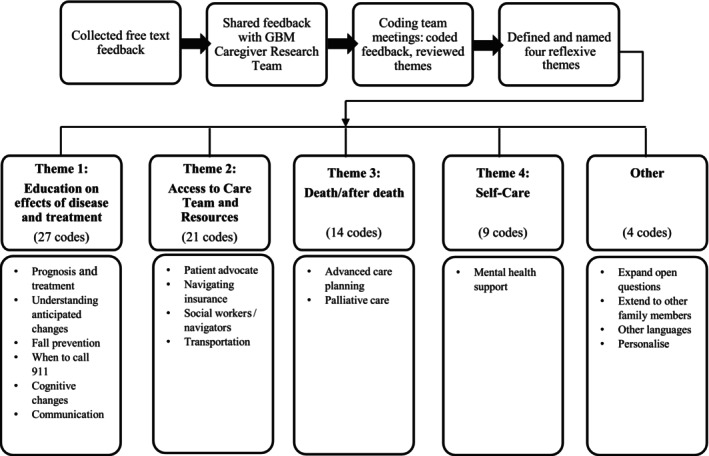
PATH feedback reflexive thematic analysis and daughter codes.

For the qualitative analysis, 42 participants suggested changes to the existing PATH instrument for a preparedness assessment of GBM caregivers that consisted of 54 separate comments: (1) 18 participants provided suggestions to revise existing questions, (2) 12 participants provided suggestions for new questions and (3) 12 participants responded to both questions. One comment could include multiple codes. The research team analysed comments and assigned 17 codes: prognosis and treatment, understanding anticipated changes, fall prevention, when to call 911, cognitive changes, communication, patient advocate, navigating insurance, social workers/navigators, transportation, advanced care planning, palliative care, mental health support, expand open questions, extend to other family members, other languages and personalise. Thirteen of the 17 codes were distilled into four themes: (1) education on effects of illness and treatment, (2) access to care team and resources, (3) death and after death and (4) self‐care. An ‘other’ section captured the remaining four codes that did not align with the four reflexive themes (Figure 6).

The codes captured in the ‘other’ section primarily reflected respondents' interest in customising the PATH instrument for GBM such as using illness‐specific terminology to ensure the patient and caregiver better understand the question. Other feedback suggested that the administration of the instrument should be extended to other family members, citing that the family was ‘on board’ with the treatment and decision‐making early in the process but sometimes less so later on. This change and/or disconnect between family members involved in care decision‐making could be because of family members' lack of understanding and need to be involved in education and care decisions throughout the illness trajectory.

#### 5.5.1 Caregiver Unmet Need Theme 1: Education on Effects of Illness and Treatment

Of the 75 codes assigned to the open‐ended feedback, those associated with education on illness and treatment effects appeared in 27 comments. Caregivers overwhelmingly expressed a lack of understanding of the diagnosis or what they and their loved one would face as a result of illness and the treatment, with 12 of the 27 comments coded to this theme reflecting this information/knowledge gap. Many caregivers reported that they were not told what to expect following diagnosis, after surgery and throughout the illness trajectory.

Caregivers did not understand the extreme changes that would take place with their loved one following diagnosis. Themes across comments suggested that medical information was not shared in a way that a non‐medical professional could understand. Four of the 27 comments coded to this theme expressed that caregivers felt that false hope was given following diagnosis and in the acute illness trajectory time period and wished that a more accurate description of what to expect was given to them.

One thing that stands out for me, was after surgery my husband not having the ability to comprehend simple conversations. Or he would obsess about certain thoughts or tasks. I learned very quickly I could no longer talk to my husband about life matters. So I guess a question in regards to awareness of what surgery can do to the brain, a more realistic answer. (GBMPATH_96)

Over one‐fourth of the comments coded to this theme indicated that caregivers had to figure out how to care for a loved one on their own, that they were not equipped with needed education or knowledge. Additional support between visits is needed, adding periodic check‐in calls and other communication methods between the caregiver and care team are desired.

No one talked to me or us as to what we would be facing from the surgery or beyond. It was fully a learn‐as‐you‐go experience and the medical team was focused on his care and survival and the presumption that I got it covered completely when we leave the building and go home – no one was asking or checking in on me from a medical team perspective. (GBMPATH_24)

The general feeling of ‘not knowing what they do not know’ was captured in the open‐ended comments and confirms similar themes that have been well documented in prior research (Coman et al. 2024). The educational gaps led to frustration and caregivers' feelings of being unprepared.

#### 5.5.2 Caregiver Unmet Need Theme 2: Access to Care Teams and Resources

Codes associated with access to care teams and resources appeared in 22 comments. Caregivers shared frustration about not being prepared when they returned home, not having better instruction on what supplies were needed, and frustration with insurance and access to physical resources for their loved one's care. Caregivers expressed fear for their loved one's safety transporting them to and from appointments.

All of my bathroom facilities are on the 1st floor of my home but I did not realize a standard wheelchair would not fit through my bathroom door until I actually tried to do it. A home visit should be conducted prior to sending a patient home or prior to the illness progression requires a wheelchair. (GBMPATH_64)

Tangible needs were expressed in the comments as well as the need for support from care team members was also highlighted. Many comments expressed themes of care team members being introduced much later in the care journey, if at all. Caregivers with stronger medical knowledge were able to advocate for their loved one and ask for additional support, but this was not common among study participants.

It would have been helpful to have more of an understanding of prognosis and what options were available and how those options worked. In my case I didn't know what palliative care is and how that would have worked as an option. I was called and interacted with palliative care at the beginning of my husband's diagnosis, but I didn't understand what they were offering. I found out after my mother‐in‐law became ill (during my husband's late stages) what palliative care actually offered. I believe my husband would have preferred to go that route if we had more of an understanding. (GBMPATH_97)

Adding care team members and other tangible resources is needed to reduce fear, improve patient care and to better support the needs of the caregiver.

#### 5.5.3 Caregiver Unmet Need Theme 3: Death and After Death

Codes associated with death and after death appeared in 14 comments. Caregivers did not feel prepared for what to do when their loved one died or how to prepare for it. There were tangible needs such as making sure they had legal affairs including trusts, wills and bank accounts in order and what to do and who to call if the patient died at home.

What to do or who to call after he has passed away. (GBMPATH_161)

They also felt many emotional needs were unmet. They described balancing false hope and information needed to prepare for life after surgery and care as the patient approaches end‐of‐life.

It asks about being mentally prepared to be a caregiver but what about for the coming loss/grief. (GBMPATH_139)

The intensity of serving as a primary caregiver is replaced by grief, loss and adjusting to life without their loved one. Comments capture gaps related to the caregiver being mentally prepared for the coming loss/grief.

#### 5.5.4 Caregiver Unmet Need Theme 4: Self‐Care

Codes associated with caregiver self‐care appeared in nine comments. Themes of burnout and gaps in mental health‐related care were common across all self‐care comments, with caregivers indicating that they became patients due to either physical burnout or mental health breakdowns. Their needs were secondary to their loved ones and no one was following up with them and checking on their unmet needs.

… I didn't know what the job as a caregiver entailed so I didn't think it would impact me as much as it did. I wish someone would have told me … I became the primary caregiver … until I had a mental health breakdown. Only then, did I reach out for serious, professional help … More specific detail as what the job of a caregiver would have been beneficial. Perhaps I would have been able to make wiser choices. (GBMPATH_78)

The topic of access to mental health support for both the patient and caregiver emerged across multiple comments. As highlighted in the care team data (Figure 1), mental healthcare support was underutilised across all study participants and only reported by 17.8% (*n* = 34) of dyads. Involving this important care team resource may benefit patients and caregivers.

Questions about the availability or access to mental health support for patient and caregiver. This is a crucial part of the process. (GBMPATH_9)

Caregivers cited not understanding what the job of a caregiver entailed and not reaching out for help until after a health breakdown. The need to care for the caregiver was a recommendation across several comments. Providing tailored support to the caregiver helps them better support their loved one as a patient and may reduce or prevent the caregiver from becoming a patient through burnout, mental health breakdown or physical illness.

## 6 Discussion

Primary caregiving for a patient with GBM is demanding due to the neurological symptoms experienced by patients that require constant attention and support and the decline in physical function increasing the need for assistance with basic and instrumental activities of daily living (Applebaum et al. 2022). Although GBM is the most common type of primary brain cancer, challenges of caregiving for a patient with other brain tumours, including metastatic brain tumours, are likely similar. Patients with brain cancer rely heavily on caregivers for emotional and physical support (Applebaum et al. 2022). Assuming the role of caregiver for a loved one with GBM is sudden and stressful (Coman et al. 2024). Existing care plans and adequate support for caregivers are either not available or insufficiently tailored to the individual needs of the caregiver to adequately prepare them to support their loved one and themselves. Work by our group and others shows caregivers report a lack of information and education needed to participate in care plans, decision‐making, and providing care for their loved ones with a progressive and incurable diagnosis, contributing to negative effects on the quality of life for both the patient and the caregiver (Au et al. 2022a, 2022b; Coman et al. 2024).

Due to the low incidence of GBM, many caregivers seek information and support from informal sources such as the internet (Miller, Maurer, and Felker 2022). The caregivers often join support groups including those on social media to connect with peers going through similar experiences (Ownsworth, Goadby, and Chambers 2015). Facebook support groups can also be a place for caregivers of patients with GBM to express what they want the medical community to know about their challenges as an informal (e.g., spouse) caregiver of a patient with GBM so that care delivery models change to include family caregivers in medical decision‐making (Coman et al. 2024).

While there were no statistically significant differences across the illness phases, response trends favoured that using the PATH instrument earlier in the illness trajectory would have been more helpful to them as caregivers. Participants in this study indicated that nearly 90% of the patients with GBM received care at major medical centers and had healthcare team members from multiple supportive disciplines including social work, mental health support, patient navigation and palliative medicine. Results from this study highlight low engagement with care team resources that can play an important role in improving quality of care for patients with debilitating illness including: (1) mental health counsellors (reported in 17.8% [*n* = 34] of dyads), (2) palliative care (reported in 33.5% [*n* = 64] of dyads) and (3) nurse navigators (reported in 33% [*n* = 63] of dyads). For at least the last decade, the positive benefits of palliative care for patients with GBM and their family caregivers have been well documented to improve quality of life measures (Collins et al. 2014), yet in our study, we observe underutilisation of this important resource. Palliative care is meant to enhance a patient's current care by focusing on quality of life during chronic and life‐threatening illnesses (Davis et al. 2015). Palliative care may also help the patient and caregiver become aware of all options available for their treatment and comfort across all stages of the illness trajectory (Lutz and Green 2016; Wu et al. 2021). Since work by our group and others demonstrates the need for more education for caregivers, enhancing palliative care engagement would likely address this resource gap. A nurse navigator focusing on the clinical aspects of a patient's care can help them to navigate their care journey with a focus on quality of timely care and to assist in answering any questions the patient or caregiver may have. A nurse navigator typically works within a healthcare facility while a palliative care specialist may work across all care disciplines (Lutz and Green 2016). Results from this study indicated the PATH instrument would be helpful for all stages of the disease trajectory and represents a valuable tool to prepare and assess the needs of caregivers of patients with GBM.

The qualitative data from this study indicated a gap in caregiver knowledge around the effects of illness and treatment. Assessing the needs of caregivers with formal measures, educating caregivers about their caregiving roles, empowering caregivers to be members of the patient's cancer team and proactively assisting caregivers is particularly important in cancer care (Berry, Dalwadi, and Jacobson 2017). Family caregivers' level of mastery predicts the survival of patients with GBM (Boele et al. 2017), yet a brain cancer or GBM‐specific preparedness assessment instrument for this population does not yet exist.

Despite the well‐documented cognitive decline of patients with GBM, caregivers report that physicians and other healthcare team members interact one‐on‐one with the patient, basically eclipsing the role of the family caregiver. Caregivers also reported ‘not knowing what they didn't know’. Developed for caregivers of patients affected by stroke, the PATH instrument was designed to uncover the unmet needs of caregivers adjusting to acute brain injuries so that care plans can be tailored to the caregiver and patient needs (Camicia, Lutz, Harvath, et al. 2021; Camicia, Lutz, Joseph, et al. 2021; Camicia, Lutz, and Theodore 2023). Future studies will focus on optimising the PATH instrument for use with caregivers of patients with GBM. The PATH instrument is specifically designed to identify these unmet needs so that care plans can be tailored by healthcare team members to address existing gaps and improve caregiver preparation.

### 6.1 Strengths and Limitations of the Work

Similarity in responses across the illness stages and for participant‐rated overall helpfulness created a homogenous sample. Since this was a convenience sample that was recruited from Facebook, caregivers who do not have access to or use social media were not included and future studies should include strategies to broaden recruitment to include this group. The study targeted caregivers in the bereavement phase for the purpose of capturing experience throughout the entire disease trajectory (rather than people who are actively providing care to a patient with GBM) which may have contributed to recall bias. Several strategies were employed to detect and reduce response bias. Recall response bias was evaluated by dividing responses based on the length of time of bereavement (26 months and below vs. more than 26 months). To eliminate neutral response bias, the survey was designed without a neutral option for participants. We reduced acquiescence bias by deploying the survey online without contact by study team members. There were 67 dropouts between providing demographic information and providing feedback about the PATH instrument. The online survey format may have contributed to this loss. Sharing a physical copy of the PATH instrument may have led to a higher survey completion rate and more detailed feedback; it might have been cumbersome to switch between the two browser tabs to view the survey (Table 1), PATH instrument (Table 2/separate tab) and then return to complete the survey (Table 1 Reviewing the PATH instrument may have triggered uncomfortable memories about the GBM journey with GBM for the participants; Camicia, Lutz, Harvath, et al. 2021) We believe that this study answered our question about the instrument's helpfulness and are planning a future study that may be conducted live (via web or in‐person) to review and discuss the instrument and its potential in an interview format. The small sample size (*n* = 124 who completed the survey) impacted the ability to report statistical significance. Extending the survey collection period (beyond 3 weeks) to increase sample size was considered, but the lack of interest on the third posting suggested that additional posts may not increase participation. A further limitation of the study was that a majority of the participants identified themselves as female carers which was not entirely surprising given the higher incidence of GBM in male patients.

### 6.2 Recommendations for Further Research

Study results suggest that the instrument would benefit the population of patients with GBM and their caregivers and support further research using the PATH instrument. Improving GBM education will better prepare the patient and caregiver for illness progression and to anticipate and navigate challenges. The IDEAL (Include, Discuss, Educate, Assess, Listen) approach to focus on the caregiver as part of the discharge planning strategy has shown benefits (Wu et al. 2021). Care, treatment and discharge plans focused only on the patient overlook the importance of the caregiver and overall family unit. Better equipping caregivers will promote their increased involvement in all aspects of care (Fernandes et al. 2017; Kinyany‐Schlachter 2017). Improving access to care resources should be pursued. Study data suggest that rehabilitation, patient/nurse navigators, use of palliative care and mental health support are underutilised. Education on available resources and when to seek additional support may increase the involvement of additional care team members.

### 6.3 Implications for Policy and Practice

Supportive patient care, including at times of new diagnosis, transfers and discharges, are core competency of nursing. Empowering nursing staff to aid in discharge and transition plans should be considered along with expanding access to patient/nurse navigators to help fill some of the current gaps and serve as an advocate for the patient with GBM and caregivers. Solutions are needed to identify needs and tailor care plans for patients with GBM. Using an instrument such as the PATH can help uncover unmet needs and help the nursing staff and other members of the healthcare team tailor discharge care plans that address the needs of both the patients and their family caregivers. This study advances the understanding of how to better equip primary caregivers to support patients with GBM. Study collaborators are actively planning future research, building on the findings of this study, to explore the impact of how implementing a systematic caregiver assessment and tailoring care plans and/or interventions can improve caregiver readiness and reduce caregiver burden.

## 7 Conclusion

This patient/caregiver population needs support prioritizing early intervention to improve caregiver readiness. Study results confirm and expand upon prior research in this area. The poor prognosis associated with GBM and the helplessness of caregivers grieving their loved ones has identified highly motivated patient and caregiver populations wishing to contribute to interventions to prevent others from suffering in the future. Willingness to participate in clinical studies is evidence of this motivation (Hendrix et al. 2016; Maurer et al. 2021).

The PATH instrument helps reveal unmet needs to enable nursing staff and other care team members to tailor care plans that address the needs of both patients and their family caregivers. This study concludes with two primary recommendations: (1) deploy the PATH instrument for caregivers and patients with GBM to respond to reported gaps with education on effects of illness and treatment, access to resources, managing death and after death and self‐care and (2) work with the primary medical, social work, financial (insurance) and other resources to fill identified gaps including education about and access to rehabilitation services and palliative care. Additional resources for the home, including home health visits, could benefit the patient and ease burden for caregivers. Even at end of life, the literature widely supports the preference of patients to return home to die if given the option (Topham et al. 2022). Participants reported high participation with hospice care (90.1%, *n* = 172) but not with palliative care (33.5%, *n* = 64). Palliative care is a holistic approach that can be integrated much earlier into the treatment plan to improve care and comfort for the patient and caregiver. For example, palliative care helps caregivers prepare for coming loss/grief, next steps when a loved one passes, and how to get personal affairs in order to be better situated to respond (Camicia, Lutz, Summers, et al. 2021; Chow and Dahlin 2018).

## Author Contributions

Caregiver feedback was evaluated through a qualitative approach including involvement of a co‐author (L.A.M.) who possesses expert knowledge of the illness area, understands the role of a caregiver, is a member of the participating Facebook group and has personal experience as a prior caregiver of a late spouse with GBM. E.A.J. conducted the study, data analysis and manuscript preparation. The other authors included students (M.P.C. and M.S.W.), and the PATH developers and expert in qualitative and caregiving research (B.J.L., M.E.C.). M.P.C. and M.S.W. participated in data analysis. L.A.M. and B.J.L. participated in data analysis, manuscript writing and editing. M.E.C. assisted with manuscript editing.

## Conflicts of Interest

Barbara J. Lutz, PhD, RN, CRRN, PHNA‐BC, FAHA, FAAN and Michelle E. Camicia, PhD, RN, CRRN, CCM, NEA‐BC, FAHA, FAAN are co‐owners of PATH2Caregiving, LLC and the Preparedness Assessment for the Transition Home (PATH) instrument. No other authors have any conflicts of interest to disclose.

### Peer Review

The peer review history for this article is available at https://www.webofscience.com/api/gateway/wos/peer‐review/10.1111/jan.16420.

## Data and Statistics

The authors have checked to make sure that our submission conforms as applicable to the Journal's statistical guidelines. The statistics were checked prior to submission by an expert statistician: Matthew Peterson, PhD, FGSA, Associate Professor, Clinical Research, School of Nursing, UNCW College of Health and Human Services. Adjunct Associate Professor of Medicine, Duke University. Associate Editor, Journal of Aging and Physical Activity. Email: petersonmj@uncw.edu.

## Supporting information


Data S1.

## Data Availability

The data that support the findings of this study are available from the corresponding author upon reasonable request.
