# Supplementary figures and images for: Former Primary Caregivers of Patients With Glioblastoma Multiforme Evaluate the PATH (Preparedness Assessment for the Transition Home) Instrument
Source: J Adv Nurs. 2024 Sep 15;81(3):1583–97. doi: 10.1111/jan.16420 (PMC11810499; doi:10.1111/jan.16420)

**Supplemental 1: Survey**


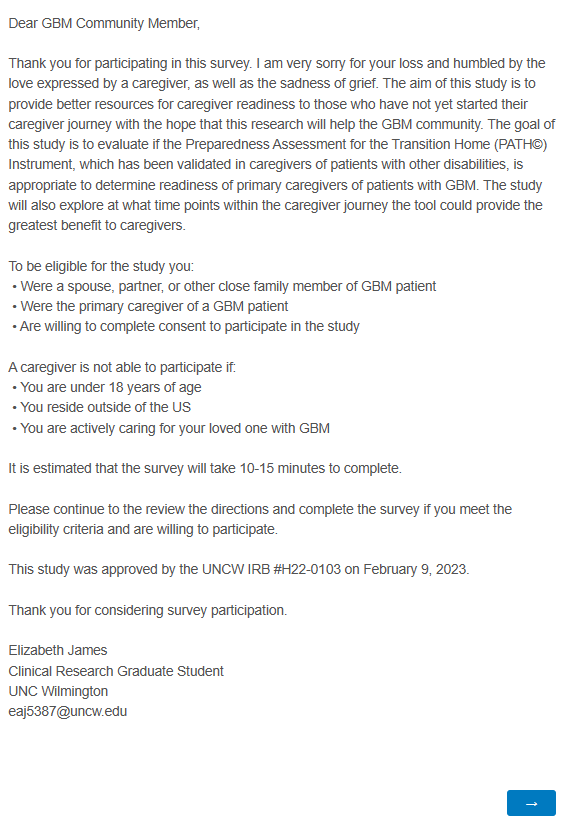


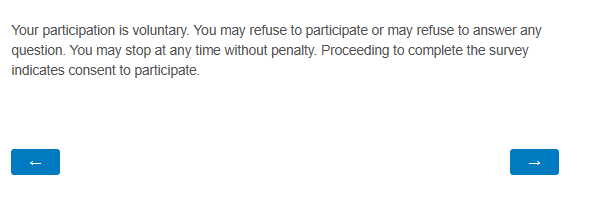


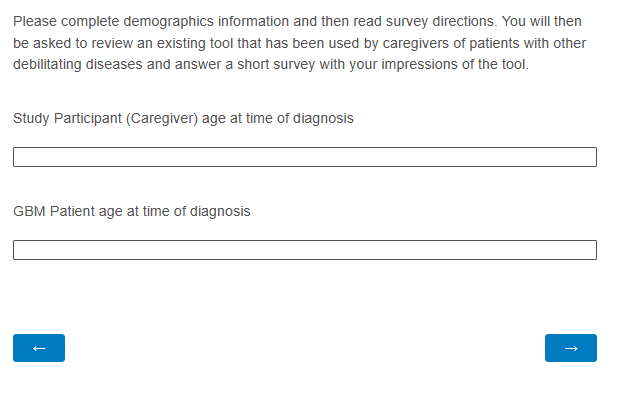


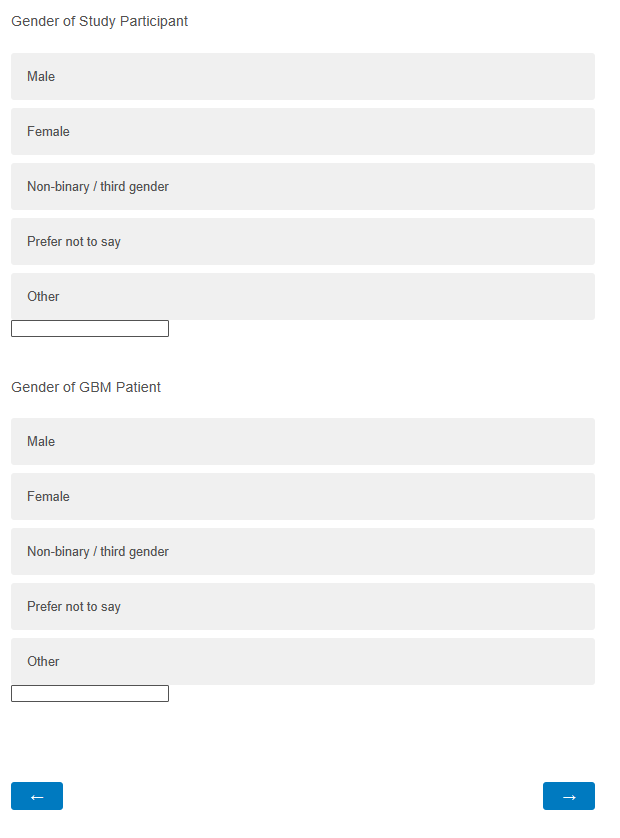

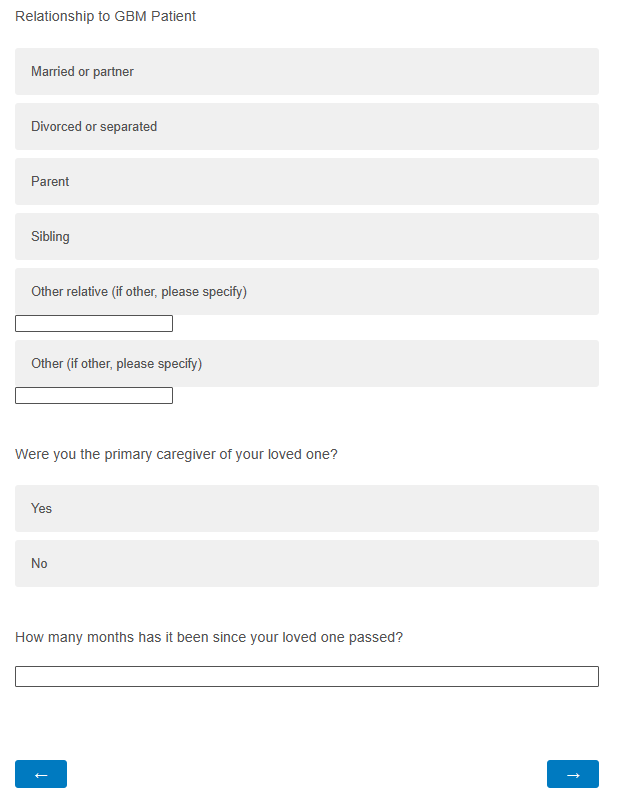


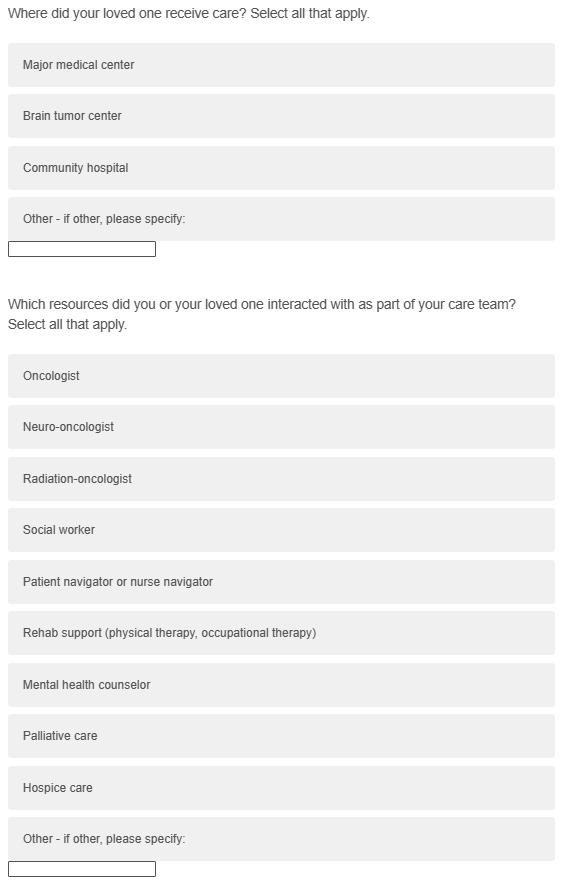


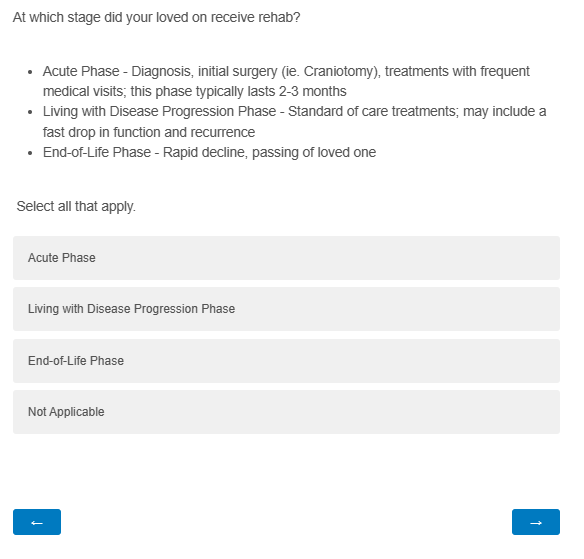


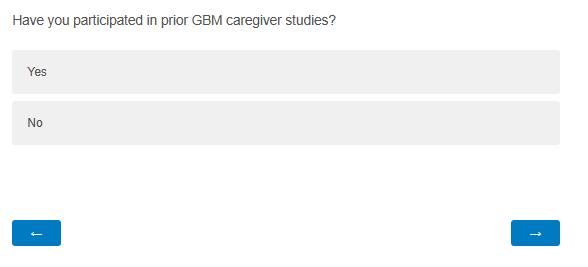

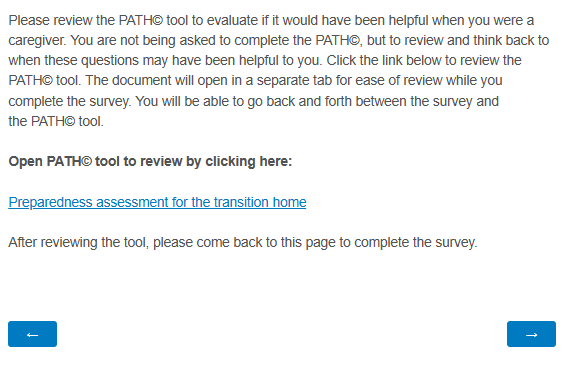

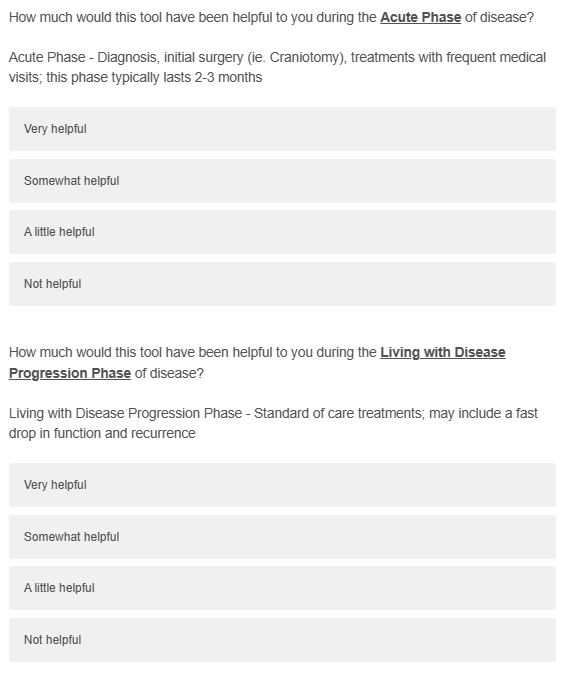

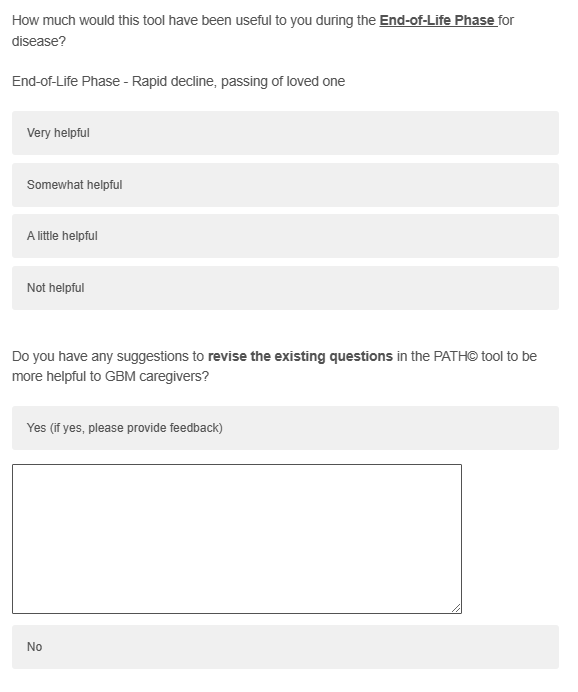

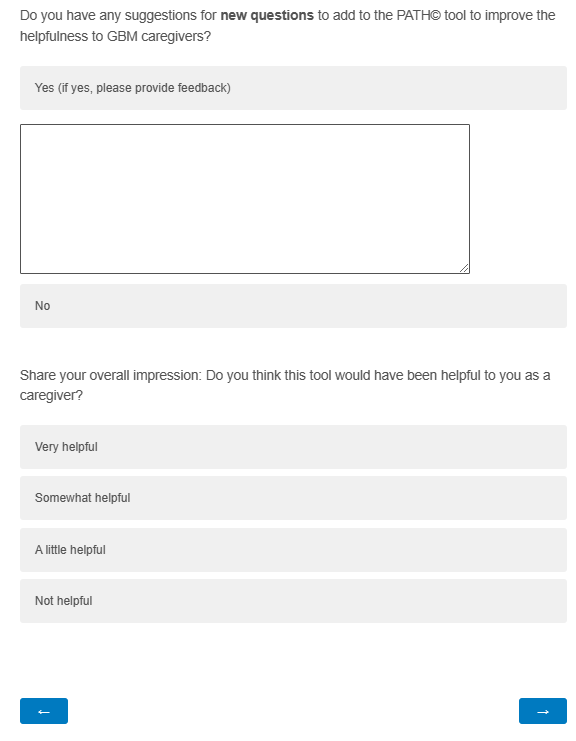

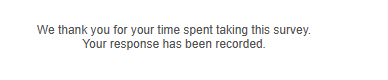

Supplement: Supplementary file 1 — Data S1. [file JAN-81-1583-s001.docx]
